# A "motor learning based intervention for lower extremities (MOBILE)" to target walking performance in ambulant children with cerebral palsy: A feasibility study.

**DOI:** 10.12688/hrbopenres.14101.1

**Published:** 2025-04-04

**Authors:** Caitríona O'Shaughnessy, Raymond McCarthy, Dereena Minehane, Jennifer Ryan, Ailish Malone

**Affiliations:** 1 Physiotherapy, CP Life Research Centre, Royal College of Surgeons in Ireland, Dublin, Leinster, D02YN77, Ireland; 2 CDNT 4 Dunshaughlin, Enable Ireland, Meath, Leinster, C15K0FV, Ireland

**Keywords:** Cerebral Palsy, Motor Learning Theory, goal-directed therapy, paediatric physiotherapy, Neurological Rehabilitation, Intensive therapy

## Abstract

**Background:**

Cerebral Palsy (CP) is the largest contributor to childhood physical disability with abnormal gait pattern such as toe walking commonly reported. The International Classification of Functioning (ICF) in disabilities framework outlines three domains to consider when looking at impact of a disability on a child; body/structure, activity and participation. Interventions to target body/structure have been widely studied but the focus is now shifting towards activity and participation. Activity and participation targeted interventions using Motor Learning Theory (MLT) have shown positive results on walking performance, gross motor skills and upper limb rehabilitation in CP. This study aims to determine feasibility and acceptability of a motor learning-based intervention for lower extremities (MOBILE) targeting walking performance in ambulant children with CP to inform a future randomized controlled trial (RCT).

**Methods:**

Fourteen ambulant children with CP, aged 6–17, with a walking goal will be recruited from community disability services. They will undergo a tailored intensive MOBILE intervention to target walking goals amounting to 30 hours practice in 6 weeks or less. Outcomes will include feasibility of recruitment, adherence, retention and outcome measures, and acceptability of the intervention. Clinical outcome measures will include the Gait Outcomes Assessment List, Six Minute Walk Test, modified Timed Up and Go, Ten metre walk test, Range of Motion and the Child Health Utility instrument. Feasibility outcomes will be reported using descriptive statistics such as percentages and confidence intervals.

**Discussion:**

Long-term retention of walking improvements in CP following interventions targeting the body/structure domain of the ICF are reportedly poor. The MOBILE intervention based on its theoretical framework could lead to improvements in walking performance with a possibility of long-term retention and impact on activity and participation. The feasibility of the study design and acceptability of the intervention needs to be investigated to inform a future definitive trial.

## Introduction

Cerebral Palsy (CP) is the largest contributor to childhood physical disability with a prevalence of 1.6/1000 births in developed countries ^1^. It is characterised by a non-progressive injury to the brain sustained prenatally or after birth up to the age of two ^2^. The sequelae in the developing child change over time and can include spasticity, muscle weakness and motor planning impairments resulting in musculoskeletal changes that impact on gross motor skills and walking ability ^3^. The extent of impairment is categorised using the Gross Motor Functional Classification System (GMFCS) which outlines the child’s level of mobility. Children at levels I-II are ambulatory without the use of aids but can have difficulties with walking endurance, speed and balance. Children at level III use an aid to walk short distances ^4^.

Gait pattern differences such as toe walking and crouched gait are prevalent in CP and walking performance deteriorates over time with reduced participation in recreational activities, increased pain and fatigue, reduced independence with self-care and reduced activity reported ^5^. Improved walking goals are therefore commonly targeted by children, parents and health professionals ^6^.

A large body of studies have targeted the body/structure domain of the ICF framework ^7^. These studies focused on surgical interventions (muscle lengthening, osteotomy), medical interventions (Botulinum Neurotoxin (BoNT), baclofen) and casting/splinting to improve range of motion (ROM) or alignment to support better walking ^8– 10^. There is some evidence these interventions improve ROM on a short term basis but only weak evidence they improve walking performance ^11^. High recurrence rates are reported for ankle contractures following BoNT, casting and surgery ^12^ and there is emerging evidence that BoNT can have a detrimental effect on the muscle tissue leading to permanent changes at the cellular level ^13^. These interventions target the ROM and alignment of the joints but improving these factors does not necessarily translate to changes in motor pattern or the skill of walking. Therefore, there is scope to explore interventions that target the source of the walking impairment and not just the resultant tissue adaptations.

Therapies that target walking include treadmill training (TT), robot assisted gait training (RAGT) and over-ground walking. A Cochrane review analysing these reported low to moderate quality evidence with a lot of variance among the studies ^14^ but acknowledged that TT and RAGT allowed for more repetitive practice and are worth further investigation. RCTs rated as good quality showed that task specificity and provision of auditory and visual feedback with gait training led to better outcomes that are clinically significant ^15^ and can have a positive impact on motor learning with larger effect sizes seen when different forms of feedback were provided during over-ground training and TT ^16^. Repetitive practice, task-specific training and provision of feedback are all elements important to motor skill acquisition according to motor learning theory ^17^.

Motor Learning Theory (MLT) encompasses a wide range of concepts and theories related to how motor skills are acquired. There are many intervention approaches which have been developed using frames of reference from MLT ^18– 20^. Interventions that use MLT in upper limb rehabilitation in CP ^18^ and for improving gross motor skills in ambulant children with CP ^21^ have been given the “green light” for use, indicating a large body of good quality evidence ^11^.

Walking is a motor skill and interventions that target walking in this population, which include motor learning concepts such as repetitive practice, task specificity and provision of feedback, are showing promise. It is therefore worth investigating if an intervention that is specifically designed to include motor learning principles to improve walking performance could be feasible. A 2023 systematic review which compiled the evidence for motor rehabilitation in children and adolescents also recommended that any interventions designed to improve a motor skill for children with CP should be goal-directed, individualised to the child, intensive, task-specific, involve repetitive practice, active use and should encourage modification of the task and environment to maximise motivation and to suit the child’s skill level ^22^. These elements are all reflected in a tool developed to support clinicians who use motor learning principles while providing interventions for upper limb function in CP ^23^. These principles and strategies have been translated into an intervention to target lower limb function with a specific focus on the motor skill of walking.

The aim of this study is to determine the feasibility of the study design and acceptability of the MOtor learning Based Intervention for Lower Extremities (MOBILE) intervention to target walking performance in ambulant children with CP.

Specific Objectives:

- Investigate the feasibility of recruitment, adherence and retention.
- Investigate the feasibility of a battery of outcome measures including time to complete, parent and child feedback and assessor experience.
- Explore the acceptability of the intervention.
- Analyse fidelity in the delivery of the program.
- Gather data to power a future definitive trial.

## Methods

### Study design

This is a single-arm prospective feasibility trial. Standard Protocol Items: Recommendations for Interventional Trials (SPIRIT) ^24^ guided the design of the protocol.

This study is registered on clinicaltrials.gov (NCT06454656) available at https://clinicaltrials.gov/study/NCT06454656?id=NCT06454656&rank=1. Last updated on August 21 ^st^ 2024 prior to enrolment. 

### Participants

Participants will be recruited from community-based, state funded, children’s therapy services (Enable Ireland).

### Eligibility criteria

A child/young person will be included in the study if they meet the following inclusion criteria and none of the exclusion criteria:

Inclusion

- Aged 6–17 with a primary diagnosis of CP (GMFCS Levels I–III)
- Has a specific walking related goal
- Has capacity to follow instruction
- Has a primary caregiver who can support a home program

Exclusion

- Has had surgery within 6 months or BoNT/baclofen within 3 months of intervention start date
- Has a dual diagnosis that impacts ability to follow instruction
- Has a significant cognitive impairment

### Recruitment strategy

Recruitment will be rolled out in stages to ensure sample size is met. Stage 1 will include 13 Enable Ireland led Children’s Disability Network Teams (CDNTs) located in Leinster (Wicklow, Dublin, Meath, Kildare, Kilkenny) and Cavan. The lead investigator will contact each team and invite them to an information evening to explain the study. Information leaflets and posters will also be sent to each CDNT. The physiotherapists will be asked to identify potential participants based on the eligibility criteria. Those identified will be provided with information leaflets and will be asked to contact the gatekeeper.

Participants may also be recruited from the specialist motor management services linked to the above-named teams. The study will also be advertised on Enable Ireland websites, CP Life Research Centre newsletter, CP Foundation Ireland newsletter and social media sites managed by Enable Ireland, CP Life Research Centre and CP Foundation Ireland.

Stage 2 will extend recruitment to the remainder of Enable Ireland led CDNTs and their linked motor management services in the rest of the country.

Informed consent will be obtained by the lead investigator in writing from eligible participants who confirm willingness to participate in the research study.

Assessments will take place in children’s services clinic in county Meath. The therapist led sessions will take place in a clinic space close to the child’s home address where possible or in an outdoor environment close to that clinic if the walking goal is specifically related to an environmental variant. This is to ensure the therapy is provided within the participant’s community.

### Sample size

For feasibility studies a sample size of 12 is recommended as it is amenable to statistical analysis being divisible by 2,3,4 and 6 and gain in precision for each increase of 1 in sample size is less pronounced when it reaches 12 ^25^. This number is rounded up to 14 to allow for 10% attrition rate.

### Intervention

The MOBILE intervention to target walking performance will be completed by all participants. The design is grounded in MLT and considers factors such as stage of motor skill acquisition, environment, feedback and types of practice ^26, 27^. The intervention design is described using a tool developed to validate the content of upper limb motor learning interventions ^23^. The full MOBILE protocol is available as extended data at https://doi.org/10.6084/m9.figshare.28524788.v1.

To determine the dose of the intervention, evidence was gathered from upper limb motor learning studies and walking-based interventions. In upper limb studies goal-directed training has been shown to lead to better gross motor outcomes than activity-based programs taking between 14 to 25 hours total practice time to achieve goals. Improvements in upper limb function have been shown to require between 30 and 40 hours of practice ^28, 29^. Interventions using treadmills or RAGT ranged from 4 to 12 weeks in duration with 2–5 sessions per week and sessions averaging 30 minutes in length which was deemed a sufficient intensity to show effect ^14^.

The MOBILE intervention will target each participants’ self-selected goal, therefore each participant will complete a minimum of 14 to 25 hours of practice. As there is limited data for improvements in gait or walking goals with MLT interventions, we will aim for each participant to perform 30 hours of practice which will allow for interruptions due to illness or other unforeseen circumstances. As the participants will be walking in their usual pattern between sessions, a massed practice format will be adopted with a minimum of two sessions per week scheduled. Therefore a maximum of 6 weeks is permitted to complete all 30 hours of practice of which a minimum of 80% will need to be supervised by the intervention therapist. These parameters were decided on following input from patient and public involvement (PPI) which was considered vital based on Faccioli’s recommendation that interventions need to consider the capacity of families and children to implement interventions ^22^. The PPI group voiced their preference for therapist led sessions in clinic versus home programs.

To target motor skill acquisition, improvement and retention, principles of neuroplasticity are embedded in the intervention design. Neuroplasticity is the brain’s adaptive capacity to encode experiences as well as learn new behaviours and skills even after brain injury. Key elements that should be considered when designing rehabilitative therapies include; intensity and specificity of practice, salience, transference and repetition ^30^.

Patient and Public Involvement (PPI) contributed to the design of this intervention. A young person and parent advisory group were consulted on intensity and frequency of sessions and preferences around home based versus clinic based sessions. This along with the evidence base in upper limb MLT studies contributed to the design of the intervention. Input from people with lived experience and the self-selected goal-targeted nature of the intervention will maximise adherence to the protocol.

Any child presenting with a plantarflexion contracture will have a heel loaded mould or cast made to allow them to experience heel contact while re-learning the motor skill of walking. This will be worn during intervention only. Plantarflexion contractures occur in 13% (GMFCS I), 24% (GMFCS II) and 46% (GMFCS III) of children with CP ^31^ which reduces their heel contact time and alters their energy use ^32^. If a child has a suitable splint or orthotic which supports heel contact they can use their own device during treatment sessions.

The intervention and casting, if required, will be delivered by the lead researcher who is a paediatric physiotherapist trained in serial casting, motor learning theory and orthotic prescription.

Any other usual care received during this time will be documented. If participants wish to discontinue the intervention, reason for dropout will be recorded.

### Outcomes

**Figure 1.**
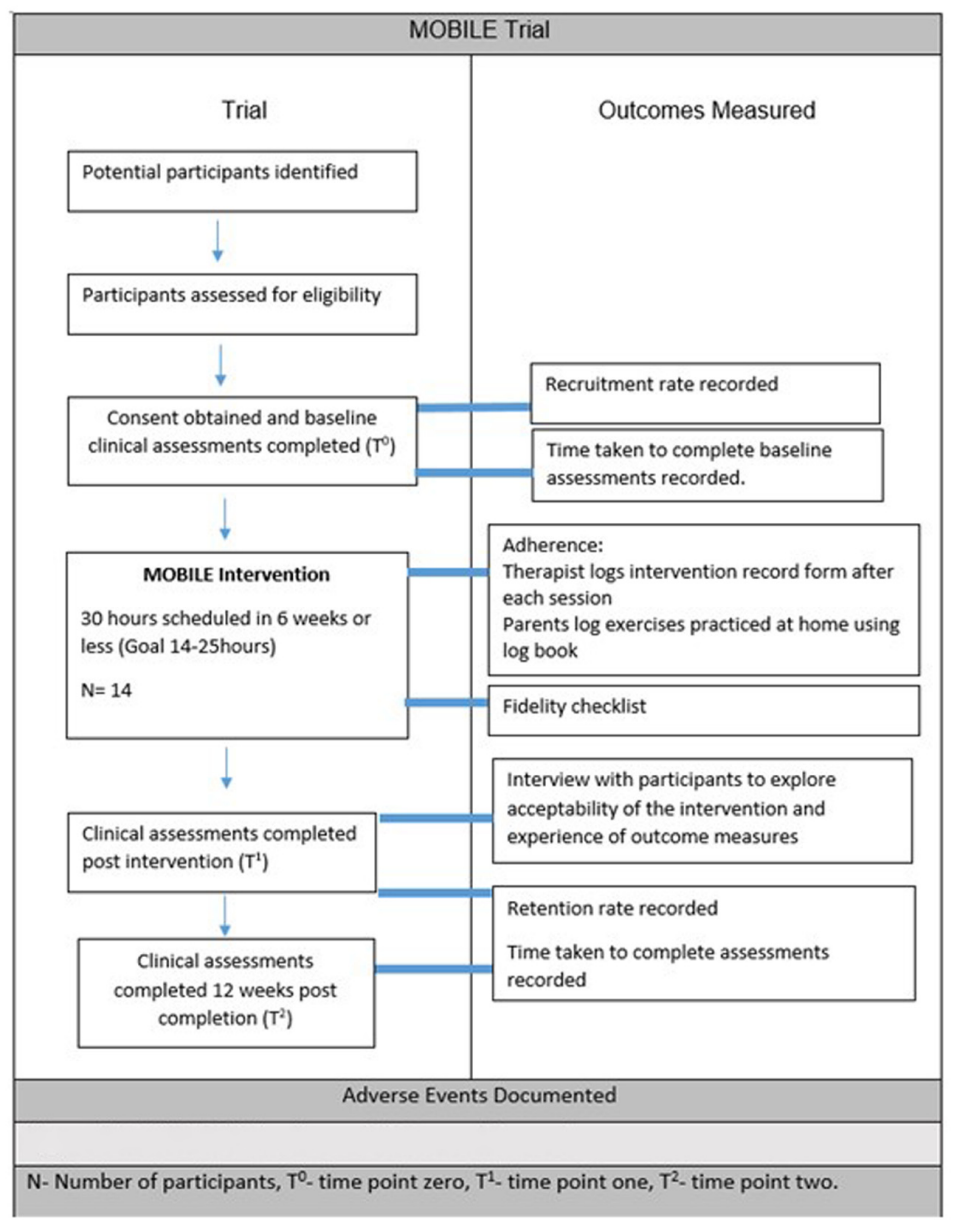
MOBILE Trial Timeline and Outcomes Measured Pg.8 Outlines the outcomes that will be measured at each timepoint throughout the study. N-Number of participants, T ^0 ^– Time point 0, T ^1^- Time point one, T ^2^-Time point 2. Attached as separate file. https://doi.org/10.6084/m9.figshare.28524986.

Outcomes will be measured at baseline (T ^0^), immediately post intervention (T ^1^) and at 12 weeks post-intervention completion (T ^2^) as outlined in Figure 1 attached as separate file (See legend at end of document). Reminders will be provided ahead of assessment dates to improve retention. All participants will be encouraged to complete all assessments regardless of completion of intervention.

***Feasibility of Study Design***

The primary outcome is feasibility of the study design with consideration given to elements that would inform on progression to RCT ^33^.

- Recruitment rates will be calculated as a percentage of eligible numbers recruited.
- Adherence will be calculated as a percentage (total hours practiced/ planned dose). Records will be kept by intervention therapist and participants to capture practice time for each session in clinic and at home.
- Retention will be calculated as a percentage of recruited participants at final follow up.
- Adverse events and serious adverse events (SAE) will be documented on relevant forms and reported to sponsor for SAE.
- Willingness to be randomized in a future trial will be reported.

***Feasibility of battery of outcome measures***

Patient and Public Involvement (PPI) informed what outcomes are important to this population with appearance of walk, fatigue/endurance, pain, how they feel about their lives and how they feel about their walk highlighted as priorities. Time it takes to complete outcome measures will be recorded for all assessments. Participant/Parent and assessor experience of outcome measures will be captured through semi structured interviews. The assessor is a physiotherapist trained in all measures who will not be involved in delivering the intervention and will also be blinded to adherence levels.

***Clinical outcome measures (T ^0^, T ^1^, T ^2^)***

The *Gait Outcomes Assessment List* (GOAL) is a parent proxy/self-report questionnaire developed to measure functional mobility and goals for ambulant children with CP. It correlates well with standard functional measures and gait analysis ^34^. It includes all domains reported by parents and young people during PPI and will be used to determine the goals for the intervention. This measure will be used in a power calculation to determine the sample size for a future definitive trial. (used with permission)

The *6 Minute Walk Test* (6MWT) is a measure of gait endurance and has been validated for use in CP ^35^. It has established Minimum Clinically Important Difference (MCID) values ^36^.

The *10 metre self-selected speed walking test* has been shown to correlate well with physical activity levels ^37^

The *Modified Timed Up and Go* (mTUG) was created to test balance and basic mobility in children with physical disabilities and has been validated for use in CP ^38^. It has established MCIDs for each level of GMFCS I-III ^39^.

*Range of Motion* of ankle dorsiflexion and knee extension will be measured using a goniometer. The Cerebral Palsy Integrated Pathway protocol for positioning will be used to standardise the measurements ^40^.

Quality of Life will be measured using the *Child Health Utilities instrument* (CHU-9D) which is a self-report measure that can calculate quality adjusted life years ^41^. (used with permission)

***Acceptability of the Intervention***

Semi-structured interviews will be conducted at the end of the intervention by a research team member not involved in delivering the intervention. These interviews, with the parent/participant, will explore the components of the Theoretical Framework of Acceptability such as self-efficacy, burden, perceived effectiveness and affective attitude ^42^. Choice of intervention frequency and mode of delivery will also be explored.

***Fidelity of the Intervention Delivery***

Fidelity of the intervention will be measured using a specific measure developed and piloted by the lead researcher based on the intervention protocol. Videos of therapist led sessions will be recorded and scored using the measure by a member of the research team not involved in the intervention, who is experienced with motor learning interventions.

## Data collection and analysis

Data will be collected on case report forms (available as extended data) and stored in a secure folder on an encrypted server. Interviews will be voice recorded and transcribed on Microsoft Teams ^TM^ using HP ProBook 450 G7 Videos will be uploaded to a Microsoft Teams folder which can only be accessed by the lead researcher and fidelity assessor.

Descriptive analysis of baseline characteristics will be carried out. These include CP type, age of child, GMFCS level, previous surgery/BoNT intervention, orthotic device worn.

The analysis will be completed in two stages. Stage one will summarise the feasibility outcomes. Recruitment, attrition and adherence rates will be reported using descriptive statistics such as percentages and 95% confidence intervals. Acceptability of the intervention and experience of the outcome measures will be described descriptively using thematic analysis of the interview transcripts.

Stage two will summarise the clinical outcomes data post intervention and 12 weeks after. As it is inappropriate to use feasibility trial data to formally test for a treatment effect, the analyses will primarily be of a descriptive nature. Interval estimates of the potential effect will be produced in the form of a 95% confidence interval and where MCIDs are reported for this population a “yes/no” will be reported if MCIDs are achieved or not.

## Discussion

The MOBILE intervention has an evidence-based design and could improve walking performance in ambulant children with CP. The protocol is in keeping with the most recent recommendations for intervention design for developing motor skills ^22^ and includes the opinions of young people with lived experience and their parents in the design. Due to the individualised and goal-targeted design, the potential benefits of the MOBILE intervention could also include longer retention of acquired motor skills which could diminish decline in functional walking skills with age and achievement of goals to support greater participation and physical activity.

Due to the time commitment and intensity of the intervention it is important to determine how feasible and acceptable it is to carry out in a community environment, embedded in practice, to inform whether a fully powered RCT could be completed to determine its efficacy. This study will inform if outcome measures such as the GOAL can be used to target a meaningful walking goal, if the participants and their parents perceive it to be effective and worthwhile and if any modifications or changes to the format of delivery could be made to improve feasibility. As there is scope for participants to choose an intensity and frequency schedule to suit their preferences, the interventionist will be able to reflect on their own experience of delivering the intervention in different formats to see if there is one that appears to be better tolerated by the participants and the therapist.

A limitation in this study is the small sample size from one regional location which may lack variety in socioeconomic backgrounds or cultural variance. This could impact the variety of data collected in interviews. Another limitation is that there is only one interventionist. This will make it challenging to determine if the protocol is easy to administer with fidelity maintained by multiple therapists across different clinics as would be needed in a multi-centre RCT. Reflexive writing will be undertaken by the intervention therapist throughout the study and discussions with wider team will be done during analysis of qualitative information to limit bias.

Patient and public involvement will be used when all data is gathered to help with interpretation of results and plan for dissemination. The final study will be published in an open access peer reviewed journal and opportunities to present at conferences will be sought.

## Ethical considerations

Ethical approval has been granted through Enable Ireland and the Royal College of Surgeons Ireland Research Ethics Committees.

Enable Ireland Research Ethics and Quality Committee (REQC) approval received on 17 ^th^ of June 2024. Reference Number: RA96

Royal College of Surgeons Research Ethics Committee approval received on the 5 ^th^ of March 2024. Reference Number: REC202312001

This study adheres to the Declaration of Helsinki for studies involving human subjects ^43^

Explicit, informed, written consent will be obtained from the parent/guardian of each participant. Written assent will also be obtained from each participant. Should a participant turn 18 during the study they will be asked to complete an updated informed written consent.

Participants will be informed of their right to withdraw at any stage without affecting their usual care. Both ethics committees will be informed of any changes to protocol via respective revision procedures.

## Protocol version control

**Table:** 

Version 1	30/06/2023	
Version 1.2	27/04/2024	Intervention Manual format change from Tidier Checklist to template for upper limb motor learning strategy tool. Change to frequency and intensity options for intervention provided based on PPI advisory panels.
Version 1.2.1	09/08/2024	Recruitment strategy expanded to include participants accessing motor management services in Enable Ireland. Primary clinical outcome measure for power calculation changed from 6minute walk test to Gait Outcome Assessment List.
Version 1.2.2	27/02/2025	Updates to background information with evidence from new systematic review added. More detail added to study limitations and discussion.

## Study sponsorship

Royal College of Surgeones Ireland Sponsorship Office- sponsorship@rcsi.ie

Maurice Dowling- Sponsor Officer

## Data Availability

No data are associated with this article. Figshare: TIDieR Description MOBILE. https://doi.org/10.6084/m9.figshare.28524953 ^44^. Figshare: MOBILE Protocol. https://doi.org/10.6084/m9.figshare.28524788 ^45^. Figshare: MOBILE Fidelity Checklist. https://doi.org/10.6084/m9.figshare.28524764 ^46^. Figshare: Data Management Plan. https://doi.org/10.6084/m9.figshare.28524650 ^47^. Figshare: Patient Information Leaflet. https://doi.org/10.6084/m9.figshare.28524803 ^48^. Figshare: Consent Form. https://doi.org/10.6084/m9.figshare.28524830 ^49^. Figshare: Case Report Form. https://doi.org/10.6084/m9.figshare.28525043 ^50^. Figshare: WHO Trial Registration Data Set. https://doi.org/10.6084/m9.figshare.28533185 ^51^. Data are available under the terms of the Creative Commons Attribution 4.0 International license (CC-BY 4.0). Figshare: SPIRIT checklist for ‘A "motor learning based intervention for lower extremities (MOBILE)" to target walking performance in ambulant children with cerebral palsy: A feasibility study’. https://doi.org/10.6084/m9.figshare.28525082 ^52^. Data are available under the terms of the Creative Commons Attribution 4.0 International license (CC-BY 4.0).
