# Supplementary material for: A "motor learning based intervention for lower extremities (MOBILE)" to target walking performance in ambulant children with cerebral palsy: A feasibility study
Source: HRB Open Res. 2026 Jan 29;8:46. Originally published 2025 Apr 4. [Version 2] doi: 10.12688/hrbopenres.14101.2 (PMC12933047; doi:10.12688/hrbopenres.14101.2)
Supplement: Peer review history

# Reviewer report: Reviewer response for version 2

**DOI:** 10.21956/hrbopenres.15751.r53254

**Published:** 2026-02-24

**Authors:** Christopher J Newman

**Affiliations:** 1 University of Lausanne, Lausanne, Switzerland

This protocol describes a pilot feasibility study of a gait oriented intervention for ambulant children with CP (GMFCS 1 to 3) based on motor-learning theories (I would avoid the singular "theory" in the text, since there is no unique motor learning theory, but the recent populae concept of "motor learning based approaches" in CP, melds a number of theoretical underpinnings).  

 This protocol added to the full intervention protocol accessible via hypertext, provides clear and sufficient information, on what the research group plans to do and how the study will be performed.

 The sample size of 12 based on one of the numerous rules of thumb from the literature on pilot studies, is arguable (see e.g. Julious 2025), but will provide sufficient insight to move forward to potential larger scale studies. I would remove the divisibility criteria since it has no pertinence to the researcher current design (no subgroups or crossovers).

Is the study design appropriate for the research question?

Yes

Is the rationale for, and objectives of, the study clearly described?

Yes

Are sufficient details of the methods provided to allow replication by others?

Yes

Are the datasets clearly presented in a useable and accessible format?

Not applicable

Reviewer Expertise:

Child neurorehabilitation

I confirm that I have read this submission and believe that I have an appropriate level of expertise to confirm that it is of an acceptable scientific standard.

## References

[1] : Sample size of 12 per group rule of thumb for a pilot study. *Pharmaceutical Statistics* .2005;4(4) : 10.1002/pst.185 287-291 10.1002/pst.185

# Reviewer report: Reviewer response for version 2

**DOI:** 10.21956/hrbopenres.15751.r53173

**Published:** 2026-01-30

**Authors:** Martin Lemay

**Affiliations:** 1 Universite du Quebec a Montreal, Montreal, Québec, Canada

I would like to thank the authors for carefully addressing my comments. I have two remaining concerns that I believe warrant further consideration.

 In your response to one of my comments, you mentioned that "This was important as the intervention relies on the participants' ability to problem solve and analyse their own movement patterns." I would recommend adding more details on this aspect, as it raises concerns that participants may rely on an internal focus of attention, which can be problematic, especially for a movement like walking that requires a certain level of automatization. For example, see this paper: Zorlular R, Akinci M, Elbasan B. Effects of focus of attention on gait parameters and balance performance in children with unilateral cerebral palsy: a randomized controlled trial. Disabil Rehabil. 2026;48(3):829-841.

 Concerning the 4th paragraph of the Introduction, while I agree that feedback is important, excessive feedback could be detrimental in some contexts, as participants might become overly reliant on it to perform the task. Therefore, it would be useful to discuss how often feedback will be provided in the intervention. For a detailed discussion on the frequency of feedback in motor learning in children with CP, see: Schoenmaker, J., Houdijk, H., Steenbergen, B., Reinders-Messelink, H. A., & Schoemaker, M. M. (2023). Effectiveness of different extrinsic feedback forms on motor learning in children with cerebral palsy: a systematic review. Disability and Rehabilitation, 45(8), 1271–1284.

Is the study design appropriate for the research question?

Yes

Is the rationale for, and objectives of, the study clearly described?

Yes

Are sufficient details of the methods provided to allow replication by others?

Yes

Are the datasets clearly presented in a useable and accessible format?

Yes

Reviewer Expertise:

Cerebral palsy; Motor learning; Adapted physical activity; rehabilitation

I confirm that I have read this submission and believe that I have an appropriate level of expertise to confirm that it is of an acceptable scientific standard, however I have significant reservations, as outlined above.

# Response

**Published:** 2026-01-30

**Authors:** Caitríona O'Shaughnessy

**Affiliations:** Physiotherapy, Royal College of Surgeons in Ireland, Dublin, Leinster, Ireland

**Thank you for your response and we are happy to further expand on your concerns.**

 In relation to Concern 1: In your response to one of my comments, you mentioned that "This was important as the intervention relies on the participants' ability to problem solve and analyse their own movement patterns." I would recommend adding more details on this aspect, as it raises concerns that participants may rely on an internal focus of attention, which can be problematic, especially for a movement like walking that requires a certain level of automatization. For example, see this paper: Zorlular R, Akinci M, Elbasan B. Effects of focus of attention on gait parameters and balance performance in children with unilateral cerebral palsy: a randomized controlled trial. Disabil Rehabil. 2026;48(3):829-841.

**Response: We agree that an internal focus of attention can be problematic if relied upon excessively. While the statement that “a participant’s ability to problem solve and analyse their own movement patterns” could imply that internal focus of attention may be used, this is not the case as planned for this intervention.**

**As you will see in the first paragraph of the intervention description, we state that the intervention “considers factors such as stage of motor skill acquisition, environment, feedback and types of practice”. In the full description of the intervention protocol listed in the extended data https://doi.org/10.6084/m9.figshare.28524788.v1 we provide a full list of the available strategies to be implemented.**

**Strategies that rely on problem solving and analysing their own movement could include ability to develop one’s own goal, participate in repetitive practice, dual-task practice, imagery practice, self-talk instructions, self-evaluation instructions, bridging settings and external focus of attention, to name a few.**

**This is a complex intervention that has many options of strategies that can be used depending on the participant’s level of skill acquisition, understanding, ability level and motivation and will be tailored by the interventionist.**

 Concern 2: Concerning the 4th paragraph of the Introduction, while I agree that feedback is important, excessive feedback could be detrimental in some contexts, as participants might become overly reliant on it to perform the task. Therefore, it would be useful to discuss how often feedback will be provided in the intervention. For a detailed discussion on the frequency of feedback in motor learning in children with CP, see: Schoenmaker, J., Houdijk, H., Steenbergen, B., Reinders-Messelink, H. A., & Schoemaker, M. M. (2023). Effectiveness of different extrinsic feedback forms on motor learning in children with cerebral palsy: a systematic review. Disability and Rehabilitation, 45(8), 1271–1284.

**Response:**

**We agree that the balance of feedback is important and have addressed this in detail in the full protocol, appended to this article as extended data https://doi.org/10.6084/m9.figshare.28524788.v1.**

**Under Principle 3: Type of Feedback, there are 9 different elements that are considered and within these 9 elements there are 33 different strategies that can be implemented as part of this intervention. As described above, this is a complex intervention that will be tailored to the individual based on their stage of skill acquisition and development.**

**The scope of feedback strategies available allow for different modes of delivery, different frequencies, different timings of delivery, the nature of the feedback and the content which, will be modified and changed as the participant progresses through the study.**

**Initially they may require error limitation and frequent feedback to ensure success and increase motivation. However, as they progress in their skill development feedback can be reduced, withdrawn or self-feedback encouraged.**

**Under the frequency element there are options for continuous feedback, faded feedback, summary feedback or bandwidth corrective feedback which can be used when the interventionist determines that this approach best suits that participant’s stage of skill development.**

**As this is a feasibility trial, it was important to include all available options for motor learning strategies and it is one of the aims of this study that the qualitative analysis of the interventionists reflective diary will provide insight into which strategies were used more frequently and why they were chosen. This will hopefully allow for refinement of the protocol to allow easier training of other interventionists in the future.**

**In response to both concerns we hope that direction towards the more detailed protocol for the intervention satisfies. Due to space constraints in the main manuscript, it was not possible to provide a full description of all the strategies that could be utilized.**

# Reviewer report: Reviewer response for version 1

**DOI:** 10.21956/hrbopenres.15494.r47068

**Published:** 2025-05-15

**Authors:** Shoaib Waqas

**Affiliations:** 1 University of Biological and Applied Sciences, Lahore, Pakistan

**Abstract**

 Could you Please include a brief mention of the theoretical framework (Motor Learning Theory) in the abstract for clarity.

**Introduction**

 Clarify the novelty of MOBILE compared to existing MLT interventions (e.g., how it differs from treadmill training or upper-limb MLT approaches).

 Briefly define "feasibility" and "acceptability" as per Sekhon’s framework (cited in methods) to align with objectives.

**Methods**

**Intervention**:

 Provide more detail on the "tailored" components (e.g., examples of walking goals or feedback strategies).

**Data Analysis**:

 Clarify how MCIDs will inform future RCT power calculations (e.g., GOAL as primary outcome).

**Discussion**

 Expand on how findings will directly inform RCT design (e.g., sample size, intervention refinements).

 Discuss potential barriers to adherence (e.g., family capacity) and mitigation strategies.

 Mention data anonymization/storage details (though covered in extended data).

**Protocol Version Control**

 Link version changes to specific limitations (e.g., expanded recruitment to address sample diversity).

**Overall Recommendations**

1. **Clarify Novelty**: Differentiate MOBILE from existing MLT interventions (e.g., task-specificity for walking vs. upper limb).
2. **Strengthen Feasibility Metrics**: Explicitly link recruitment targets (n=14) to progression criteria for RCT (e.g., ≥80% adherence).
3. **Expand Intervention Details**: Provide examples of motor learning strategies (e.g., feedback types, task modification).
4. **Highlight PPI Impact**: Show how advisory groups shaped intervention design (e.g., clinic vs. home preference.

Is the study design appropriate for the research question?

Yes

Is the rationale for, and objectives of, the study clearly described?

Yes

Are sufficient details of the methods provided to allow replication by others?

Yes

Are the datasets clearly presented in a useable and accessible format?

Yes

Reviewer Expertise:

Pediatric Physical Therapy

I confirm that I have read this submission and believe that I have an appropriate level of expertise to confirm that it is of an acceptable scientific standard, however I have significant reservations, as outlined above.

# Response

**Published:** 2026-01-26

**Authors:** Caitríona O'Shaughnessy

**Affiliations:** Physiotherapy, Royal College of Surgeons in Ireland, Dublin, Leinster, Ireland

Abstract

 Could you Please include a brief mention of the theoretical framework (Motor Learning Theory) in the abstract for clarity.

**We agree it is important to mention Motor Learning Theory. We added a brief description that MLT encompasses multiple concepts that shape our understanding of how motor skills are acquired and developed.**

Introduction

 Clarify the novelty of MOBILE compared to existing MLT interventions (e.g., how it differs from treadmill training or upper-limb MLT approaches).

**The novelty and need for MOBILE is explained in three main points.**

1. **RAGT and treadmill training, while having one or two elements that are known to influence motor learning, were not designed specifically to target motor learning (Introduction, fourth paragraph).**
2. **Upper limb and gross motor MLT approaches do not specifically focus on lower limbs or walking as the target (Introduction, paragraph 5)**
3. **There is currently a lack of interventions designed using MLT that specifically target walking as a motor skill (Introduction, final paragraph).**

Briefly define "feasibility" and "acceptability" as per Sekhon’s framework (cited in methods) to align with objectives.

**Definitions of feasibility and acceptability have been added to the section preceding the objectives as suggested.**

Methods

Intervention:

 Provide more detail on the "tailored" components (e.g., examples of walking goals or feedback strategies).

**Three new paragraphs (paragraphs 4-6) have been added to the intervention section providing more detail on the tailored components of the intervention. Some examples of walking goals, feedback strategies and other factors are provided. It has also been added that the intervention therapist will keep a reflective diary throughout which will document how each session developed and why tasks were chosen. This will serve as the basis for developing an instruction manual for other therapists wishing to replicate the intervention.**

Data Analysis:

 Clarify how MCIDs will inform future RCT power calculations (e.g., GOAL as primary outcome).

**We have expanded the final paragraph of the data analysis subsection to clarify that MCIDs are only being used to estimate a potential effect in the two measures where there are established MCIDs for this population. The GOAL was selected as the primary clinical outcome measure by the PPI advisory group as it was the most meaningful measure to them. There currently are no established MCIDs for the GOAL therefore a power calculation will be done on the data collected for the total GOAL score.**

Discussion

 Expand on how findings will directly inform RCT design (e.g., sample size, intervention refinements).

**The third and fourth paragraphs of the discussion section have been expanded to provide more detail on how the results will inform an RCT design including (1) potential need for a multi-centre design will be judged on time for recruitment and geographical spread, (2) balance of clinic and home-based programs will be judged on qualitative feedback from interviews and adherence rates and (3) intensity and frequency or other intervention design refinements will be considered based on qualitative analysis from the interviews and feasibility metrics**

**A fifth paragraph has been added to further discuss how the information collected by the interventionist will help to refine the intervention manual.**

 Discuss potential barriers to adherence (e.g., family capacity) and mitigation strategies.

**The second paragraph of the discussion has been expanded to discuss potential barriers to adherence and mitigating strategies. Briefly, these include participant fatigue or competing demands with school or hobbies, balancing appointments and home programs with other family demands, and physical fatigue or muscle soreness. Rest breaks will be given as needed, appointments will be scheduled to suit families as best possible and home programs will be simple and incorporated into their hobbies or other activities where possible.**

 Mention data anonymization/storage details (though covered in extended data).

**Brief mention of anonymisation and storage has been added to the first paragraph of the “data collection and analysis” subsection, which now explains that anonymised data will be uploaded to a secure SharePoint folder. We also direct readers to our data management plan under extended data.**

Protocol Version Control

 Link version changes to specific limitations (e.g., expanded recruitment to address sample diversity).

**Linked version change 1.2.1 expansion of recruitment strategy to address more diversity in sample size as suggested. Other changes were due to new evidence emerging or feedback from PPI groups rather than limitations prompting changes.**

Overall Recommendations

 1. Clarify Novelty: Differentiate MOBILE from existing MLT interventions (e.g., task-specificity for walking vs. upper limb).

**Addressed above in the introduction.**

 2. Strengthen Feasibility Metrics: Explicitly link recruitment targets (n=14) to progression criteria for RCT (e.g., ≥80% adherence).

**Specific feasibility metrics for progression were not included for this study as there will likely be many refinements and adjustments to the protocol based on an integrated analysis of the quantitative and qualitative data collected. This is described in an addition to the first line under “Feasibility of Study Design” in the Outcome section of the methodology, It is also added that PPI groups will be involved in the decision-making process around recommendations on progression to RCT.**

 3. Expand Intervention Details: Provide examples of motor learning strategies (e.g., feedback types, task modification).

**Addressed above in detail.**

 4. Highlight PPI Impact: Show how advisory groups shaped intervention design (e.g., clinic vs. home preference.

**More detail was added to the PPI paragraph and are highlighted in the comments to reviewer 1.**

# Reviewer report: Reviewer response for version 1

**DOI:** 10.21956/hrbopenres.15494.r46650

**Published:** 2025-05-08

**Authors:** Martin Lemay

**Affiliations:** 1 Universite du Quebec a Montreal, Montreal, Québec, Canada

This single-arm feasibility trial evaluates the MOBILE intervention to improve walking in children with cerebral palsy aged 6-17 (GMFCS Levels I-III). Participants will receive 14-30 hours of therapy over 6 weeks, targeting self-selected walking goals using motor learning principles. Feasibility outcomes like recruitment, adherence, and retention will be assessed, along with clinical outcomes such as gait endurance, mobility, and quality of life. The study incorporates patient and public involvement (PPI) to ensure the intervention meets participants' needs. This well-thought-out protocol, however, relies on a relatively small sample size and should better consider the heterogeneity of CP.

 Rationale and objectives

- The introduction refers only to children with GMFCS levels I to III, but it is important to acknowledge that CP also includes children classified at levels IV and V. While these individuals are typically non-ambulant and may not be the primary focus of this study, briefly recognizing their existence and the broader spectrum of motor impairments within CP would provide a more complete overview of the condition.
- I would consider explicitly articulating the specific gap this feasibility study aims to address—for example, the current lack of motor learning–based interventions specifically tailored to improve lower limb function in ambulant children with cerebral palsy.
- The study objectives are clearly articulated and appropriate for a feasibility study. They effectively lay the groundwork for a future investigation into the efficacy of the MOBILE intervention.

Study design

- CP is highly heterogeneous, with significant variability in severity and associated conditions. How can you account for these differences in your study design and methodology to ensure that your findings are applicable to all children with CP, regardless of their specific diagnosis or functional level?  This is especially important in the context of a study involving only 14 participants across a broad age range, where individual differences may play a crucial role in the outcomes.  Also, while the multi-stage recruitment strategy is well thought out, it relies heavily on therapist referrals and might not capture a diverse enough sample.
- It is indicated that consent will be obtained. Both consent (from the guardian) and assent (from the child) should typically be obtained when conducting research with children, with the assent process tailored to the child's age and level of understanding
- The rationale for the chosen dose of the intervention is well-justified
- The involvement of a young person and parent advisory group in shaping the intervention and providing input on session intensity, frequency, and mode of delivery enhances the relevance and acceptability of the intervention for the target population. However,more details on the advisory group consultation are needed, including the number of participants, the frequency of meetings, and the specific nature of their contributions.
- While the clinical outcome measures appear relevant, it would be useful to provide a brief rationale for their inclusion.

Replication

- The protocol follows the SPIRIT guidelines, which is a well-established standard for clinical trials. This provides a solid framework for replication. The original SPIRIT 2013 guidelines have undergone updates and refinements in recent years. Have these more recent developments been taken into account in the preparation of this protocol?
- More details on the categorization of participants in terms of age, GMFCS level, comorbidities, type of walking, and other relevant factors are needed.
- The “ *Fidelity of the Intervention Delivery*” section provides a clear overview of the actions taken to ensure consistent provision of the intervention across therapists. However, it is important to include more information on the therapist, its training and experience, and other relevant factors that may impact the delivery of the intervention.

Is the study design appropriate for the research question?

Yes

Is the rationale for, and objectives of, the study clearly described?

Yes

Are sufficient details of the methods provided to allow replication by others?

Yes

Are the datasets clearly presented in a useable and accessible format?

Yes

Reviewer Expertise:

Cerebral palsy; Motor learning; Adapted physical activity; rehabilitation

I confirm that I have read this submission and believe that I have an appropriate level of expertise to confirm that it is of an acceptable scientific standard, however I have significant reservations, as outlined above.

# Response

**Published:** 2026-01-26

**Authors:** Caitríona O'Shaughnessy

**Affiliations:** Physiotherapy, Royal College of Surgeons in Ireland, Dublin, Leinster, Ireland

Rationale and objectives

- The introduction refers only to children with GMFCS levels I to III, but it is important to acknowledge that CP also includes children classified at levels IV and V. While these individuals are typically non-ambulant and may not be the primary focus of this study, briefly recognizing their existence and the broader spectrum of motor impairments within CP would provide a more complete overview of the condition.

**I have expanded the description of the GMFCS levels to include those at level IV and V as suggested. This description is at the end of paragraph one of the introduction and describes why levels I to III were chosen with the focus of the study targeting children who walk functionally with independence.**

- I would consider explicitly articulating the specific gap this feasibility study aims to address—for example, the current lack of motor learning–based interventions specifically tailored to improve lower limb function in ambulant children with cerebral palsy.

**In the final paragraph of the introduction, before the aims, I describe the research currently published that includes concepts of motor learning theory to target walking which include task specificity, repetitive practice and provision of feedback. I have added a sentence that explicitly states the lack of motor learning-based interventions to target lower limbs for walking goals as correctly articulated by the reviewer.**

**Also in response to reviewer two, more detail was added to introduction paragraphs four and five describing the interventions in the current evidence base which adds to the gap this study aims to address.**

Study Design

- CP is highly heterogeneous, with significant variability in severity and associated conditions. How can you account for these differences in your study design and methodology to ensure that your findings are applicable to all children with CP, regardless of their specific diagnosis or functional level?  This is especially important in the context of a study involving only 14 participants across a broad age range, where individual differences may play a crucial role in the outcomes.  Also, while the multi-stage recruitment strategy is well thought out, it relies heavily on therapist referrals and might not capture a diverse enough sample

**For this study, the focus is on a very specific cohort within the CP population. Due to the inclusion and exclusion criteria only children/ young people with good cognitive ability and no other co-morbidities that would impact on their ability to concentrate are eligible for this particular intervention. This was important as the intervention relies on the participants ability to problem solve and analyse their own movement patterns. In terms of the age range and different physical presentations within the eligible cohort the interviews will aim to capture where individual differences may impact on the outcomes. Ideally a larger cohort of participants would be preferred however the number had to be feasible within the constraints of this study’s particular timeline and resources. Also, due to Ireland’s population size, the number of eligible participants in the area that would be feasible to travel for this intervention would not be large. If a multi-centre design was being used in different locations, a larger number of participants would be more feasible and this is something we would consider for future work, with appropriate funding. In relation to the recruitment strategy, in Ireland, all children with a physical disability are linked to their local public team and will be known to their local therapist but advertisement of the study is being done through other avenues including CP advocacy groups to ensure all interested potential participants have an opportunity to engage with the research team, even if their therapist does not refer them directly.**

- It is indicated that consent will be obtained. Both consent (from the guardian) and assent (from the child) should typically be obtained when conducting research with children, with the assent process tailored to the child's age and level of understanding

**Both assent and consent will be obtained. In the second to last paragraph under the subheading “recruitment strategy” where I discuss Informed consent procedure, I have added the information regarding participant assent also and have included a figshare link to the assent form in the extended data section**

- The involvement of a young person and parent advisory group in shaping the intervention and providing input on session intensity, frequency, and mode of delivery enhances the relevance and acceptability of the intervention for the target population. However,more details on the advisory group consultation are needed, including the number of participants, the frequency of meetings, and the specific nature of their contributions.

**The paragraph discussing the PPI contributions in the intervention description section now contains more detail in relation to the young person and parent advisory group as suggested. It now describes how 5 young people aged 10 to 17 and 12 parents contributed to either a half-day in-person session or a 2 hour online session to discuss the intervention design and outcome measures.**

  

- While the clinical outcome measures appear relevant, it would be useful to provide a brief rationale for their inclusion.

**Where the clinical outcome measures are listed I adjusted the phrasing for the 6 minute walk test, 10 metre self-selected speed walking test and the modified timed up and go test to explain that these three measures were selected due to their previous use in related studies and have either shown good correlation to physical activity levels which is important under the ICF guidelines or they have established MCIDs for this population which can give an indication as to whether any meaningful difference was achieved by the participants. I also added further description to rationalise using range of motion and quality of life measures which includes recognizing that contractures and surgical interventions are common in this population and quality of life measures can capture the overall impact an intervention has on an individual.**

Replication

- The protocol follows the SPIRIT guidelines, which is a well-established standard for clinical trials. This provides a solid framework for replication. The original SPIRIT 2013 guidelines have undergone updates and refinements in recent years. Have these more recent developments been taken into account in the preparation of this protocol?

** At the time of creating and publishing this protocol the SPIRIT 2013 checklist was the most up to date version and is referenced below the extended data section with a link to a figshare document. I have added “2013” in the study design section to highlight that this was the version used. The newest version SPIRIT 2025 was published this year and contains an extra open science section, requires more information regarding comparators and harms and a section on patient and public involvement.  I have reviewed the newest checklist and the main changes that are relevant to my protocol are the open science section and the PPI section. I am confident that my protocol contains the information recommended by the 2025 guideline for both additional items on the checklist.**

  

- More details on the categorization of participants in terms of age, GMFCS level, comorbidities, type of walking, and other relevant factors are needed.

**The inclusion and exclusion criteria outline the age range, GMFCS level and comorbidities that will be expected. Comorbidities such as any condition that impacts cognition and attention are excluded.**

**In the data collection and analysis section I included, body distribution and gait aids used to provide more detail in terms of type of walking that will be described descriptively.**

**I added rationale for not categorizing participants based off baseline characteristics for analysis due to the small sample size but state that the interviews will allow exploration of any challenges or experiences that may have been impacted by factors such as age or GMFCS level.**

**I also added a paragraph to the intervention description describing how each session will be tailored to a participant based on the categories described by the reviewer. The intervention will be adapted to each child therefore there will be no specific tasks practiced based off a participant’s walking ability or age and therefore further categorization will not add any extra value. The intervention therapist will keep a reflexive diary to show how tasks were adapted based of these characteristics.**

  

- The “ *Fidelity of the Intervention Delivery*” section provides a clear overview of the actions taken to ensure consistent provision of the intervention across therapists. However, it is important to include more information on the therapist, its training and experience, and other relevant factors that may impact the delivery of the intervention.

**Further details have been provided on the fidelity assessor and intervention therapist in relation to experience level and training for both. This can be found under the subheading “fidelity of the intervention delivery” and describes the fidelity assessor as an occupational therapist with extensive experience in upper limb motor learning therapies and the interventionist as a physiotherapist with experience in supporting children with childhood onset disabilities with further training completed in upper limb motor learning interventions and certificate in a new online motor learning training platform.**
